# Artificial Neural Network Modeling of Ti-6Al-4V Alloys to Correlate Their Microstructure and Mechanical Properties

**DOI:** 10.3390/ma18051099

**Published:** 2025-02-28

**Authors:** Anoop Kumar Maurya, Pasupuleti Lakshmi Narayana, Jong-Taek Yeom, Jae-Keun Hong, Nagireddy Gari Subba Reddy

**Affiliations:** 1 Lightweight Materials Research Division, Korea Institute of Materials Science, Changwon 51508, Republic of Koreajkhong@kims.re.kr (J.-K.H.); 2 Virtual Materials Laboratory, School of Materials Science and Engineering, Engineering Research Institute, Gyeongsang National University, Jinju 52828, Republic of Korea

**Keywords:** artificial neural network (ANN), mechanical properties of Ti-6Al-4V alloy, index of relative importance, weight distribution, sigmoid activation function

## Abstract

The heat treatment process of Ti-6Al-4V alloy alters its microstructural features such as prior-β grain size, Widmanstatten α lath thickness, Widmanstatten α volume fraction, grain boundary α lath thickness, total α volume fraction, α colony size, and α platelet length. These microstructural features affect the material’s mechanical properties (UTS, YS, and %EL). The relationship between microstructural features and mechanical properties is very complex and non-linear. To understand these relationships, we developed an artificial neural network (ANN) model using experimental datasets. The microstructural features are used as input parameters to feed the model and the mechanical properties (UTS, YS, and %EL) are the output parameters. The influence of microstructural parameters was investigated by the index of relative importance (IRI). The mean edge length, colony scale factor, α lath thickness, and volume fraction affect UTS more. The model-predicted results show that the UTS of Ti-6Al-4V decreases with the increase in prior β grain size, Widmanstatten α lath thickness, grain boundaries α thickness, colony scale factor, and UTS increases with mean edge length.

## 1. Introduction

**Figure 1:**
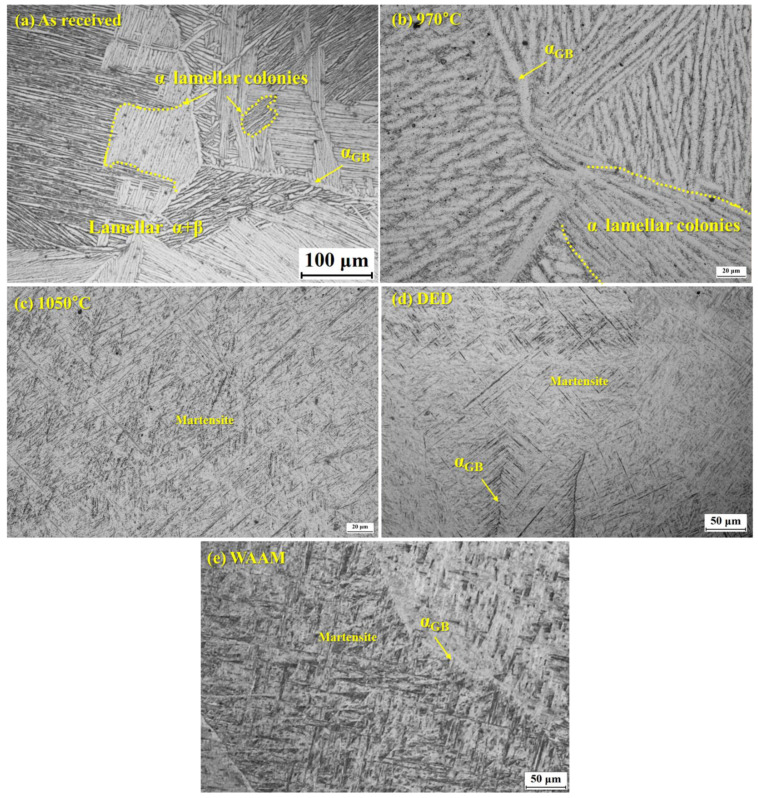
Experimental optical microstructures of conventional billet Ti-6Al-4V. (**a**) As received, (**b**) 970 °C-1 h/WQ, (**c**) 1050 °C-1 h/WQ, and (**d**) DED. (**e**) WAAM Ti6Al4V.

Ti-6Al-4V alloys are mainly used in aerospace, biomedical, marine, and structural applications due to their high strength-to-weight ratio, fracture toughness, elastic modulus, and high corrosion resistance. The manufacturing technology and microstructure variation in traditional titanium alloys have restricted the application of Ti-6Al-4V alloys for specific applications [1]. The conventional techniques provide a route to manufacture complex and large parts such as aircraft engine frames, fan frames, compressor casings, exhaust gas pipes of auxiliary gas turbines, and rim screws [2]. Components of Ti-6Al-4V are generally produced by hot working steps and followed by heat treatment. The heat treatment of a Ti-6Al-4V component is performed based on service requirements [3]. Recently, the additive manufacturing technique (AM) [4,5,6] has been applied to produce complex and near-net-shape Ti-6Al-4V components through layer-by-layer deposition, but such manufactured AM components need further heat treatment to modify the microstructure according to the application [7,8,9]. The control of microstructural evolution is important to control the physical and mechanical properties. In conventional melting techniques, the beta grain size lies between 2 and 5 mm [10]. The mechanical properties are the important criteria of Ti-6Al-4V component service capabilities in aerospace and other industrial applications. The microstructure is an important factor of the alloy, controlling its tensile properties, fracture toughness, and fatigue strength. The microstructure of Ti-6Al-4V varies over a wide range, depending on heat treatments or thermomechanical processes and manufacturing techniques [11,12,13,14]. It has been also found that a solution treatment close to the transformation microstructure can significantly alter the wear resistance of a Ti-6Al-4V component due to changes in microstructure [15]. A solution treatment at high temperature in the α+β region or β phase region, followed by long aging periods, increases tensile strength and hardness but with the loss of ductility and impact toughness [16,17]. However, annealing in the α+β region for a longer time, followed by air or furnace cooling, improves the ductility and impact toughness with a loss of tensile strength and hardness [18]. The heat treatment of Titanium alloys controls the morphology, volume fraction of α and β phases, secondary α-phase, prior-β grain size, Widmanstatten α lath thickness, Widmanstatten α volume fraction, grain boundary α thickness, α colony size, and α platelet length. Generally, two distinct microstructures can be produced in Ti alloys depending on the undercooling of the β phase field. When the Ti alloy is cooled at low rates from the β phase, the α phase first nucleates at the β grain boundaries, which is incoherent with respect to the β phase and leads to more or less continuous α layer along the β grain boundaries called grain boundary α (α_GB_). With continuous cooling, the α further nucleate at the α_GB_ or at the prior β grain boundaries itself and grow inside the grain as parallel plates as an α colony until they meet another α colony nucleated at the other side of the grain boundaries. The α plates within one colony belong to the same Burgers orientation relationship (BOR). This process is called sympathetic nucleation and growth, and the resultant microstructure is referred to as a lamellar microstructure [19]. With a large undercooling, a basket weave microstructure is produced, where a high thermodynamic driving force, favors the intragranular nucleation and growth of multiple variants of α [20]. Stefansson et al. [21] introduced the mechanism of the statics globalization of the lamellae of Ti-6Al-4V through deformation and annealing. For example, the microstructure of Ti-6Al-4V obtained from the conventional manufacturing technique (β-forged billet) is shown in Figure 1a and exhibits a lamellar microstructure with α colonies. The few α colonies are highlighted with dotted yellow lines (Figure 1a). The heat treatment just below the β transus temperature (990 °C) at 970 °C for 1 h followed by water quenching (WQ) results in the formation of coarse α lamellae (Figure 1c). In contrast, the microstructure heat-treated above the β transus temperature (980 °C) at 1020 °C for 1 h with WQ represents the formation of martensite containing very fine α lamellae (Figure 1c). The microstructures of additively manufactured Ti-6Al-4V using Wire Arc Additive Manufacturing (WAAM) and Direct Energy Deposition (DED) are shown in Figure 1d and e, respectively. The prior β grain size and α-lath thickness differ between both processes, which influences the mechanical properties [22].

The microstructure of Ti-6Al-4V mainly depends on the processing parameters of the used technique and heat treatment, which affect the chemical and physical properties of alloys [23]. The change in the heat treatment temperature impacts the amount of beta phase fraction, which affects the mechanical properties. The higher the heat treatment temperature, the more beta phase is present and trapped during water quenching. During aging, the retained beta can transform to produce a bimodal alpha distribution [12,24]. Gil Mur et al. [25] investigated the decomposition of α’ phase via heat treatment in the temperature range of 400 °C–800 °C and found that temperatures higher than 700 °C result in complete decomposition of α’; however, below 600 °C, it remained incomplete. The aspect ratio of alpha platelets and grain size significantly affect the mechanical properties of Ti-6Al-4V alloy components. It was found that a low aspect ratio of alpha platelets shows high fatigue-crack propagation resistance, high fracture toughness, and improved creep strength [26]. The globular alpha and equiaxed alpha, which form after the thermomechanical treatment process, show high strength and percentage elongation [27]. It has also been found that the heat treatment temperature for stress relief also improves the ductility of Ti alloys [28].

Ti-6Al-4V alloys are widely used in the aerospace, biomedical, and marine sectors, yet their performance in specialized applications is limited by microstructural variations resulting from conventional and additive manufacturing (AM) techniques. While heat treatment effects on microstructural features such as α lath thickness, β grain size, and α colony size have been studied, their precise and quantitative impact on mechanical properties—such as ultimate tensile strength (UTS), yield strength (YS), and elongation (%EL)—remains unclear. Additionally, the influence of microstructural heterogeneity on fatigue, creep, and wear resistance is insufficiently understood.

The objective of this research is to determine the relationship between microstructural features (e.g., prior-β grain size, α lath thickness, α colony size, and volume fractions) and mechanical properties (UTS, YS, %EL) of the Ti-6Al-4V alloy. To establish this relation, we develop an artificial neural network (ANN) model to analyze and predict the complex, non-linear relationships between heat treatment parameters, microstructural features, and mechanical properties. This study also provides insights for optimizing heat treatment strategies to enhance the performance of Ti-6Al-4V alloys in conventional and AM applications.

## 2. Materials and Methods

### 2.1. Data Collection and Input–Output Variables of the Model

An ANN model is a computational program used to solve very complex and non-linear problems in various fields of materials science and engineering [29,30,31]. An ANN model contains neurons connected with a coefficient called weights. Each neuron connects to the others and forms a network called a neural network. The power of neurons comes from connecting weights in the network. This is similar to how biological neurons connect and form a long neural network. An ANN model gathers knowledge by detecting the relationship between experimental datasets, this phenomenon is called training and testing of the model. The model accuracy depends on the minimum error in prediction during training.

The mechanical properties of Ti-6Al-4V alloys were determined through standard tensile testing procedures following established ASTM guidelines. The tests were conducted at room temperature, with samples prepared and tested under controlled conditions to ensure consistency. Ultimate tensile strength (UTS), yield strength (YS), and elongation (%El) were measured from stress–strain curves obtained during the tests. The specimens were subjected to uniform loading conditions to accurately capture their mechanical response. The data were then grouped based on heat treatment conditions and microstructural variations, ensuring that each dataset reflected distinct processing routes and corresponding mechanical behavior. To enhance reproducibility and minimize variability, multiple samples were tested for each condition, and the averaged values were used in the analysis [32].

In this model, a total of 75 experimental datasets were used, of which 55 datasets were used for training and 20 datasets were used for testing the model; the data were collected from the published literature [32].

All input and output data were normalized between 0.1 and 0.9 by using the following equation [29].

(1) $$x_{n}=\frac{x-x_{min}}{x_{max}-x_{min}}+0.1$$

where $x_{n}$ is the normalized value of x, and $x_{max}$ and $x_{min}$ are the maximum and minimum values of x, respectively, in the whole dataset. After training the model, all the datasets were again entered into the following equation [29].

(2) $$x=\frac{\left(x_{n}-0.1\right)\left(x_{max}-x_{min}\right)}{0.8}+x_{min}$$

There are a total seven input process parameters, including prior β grain size (prior β), Widmanstatten α lath thickness (Wida), Widmanstatten α volume fraction (Vfwida), Grain boundary α thickness (GB α), total α volume fraction (VFtotal), α colony size (CS) and mean edge length (MEL), as shown in Table S1 of Supplementary Material. The yield strength (YS), ultimate tensile strength (UTS), and % elongation (%El) are the output parameters in the present model.

### 2.2. Training Procedure of the ANN Model

**Figure 2:**
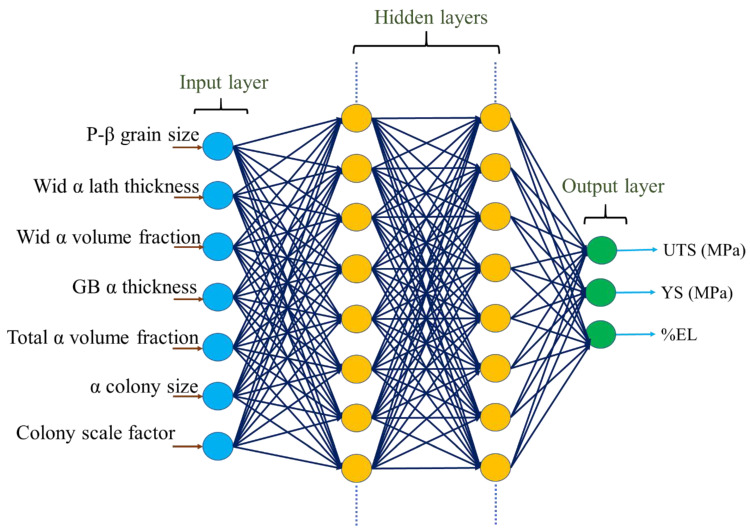
ANN model with the input layer, hidden layers with hidden neurons, and output layer.

**Figure 3:**
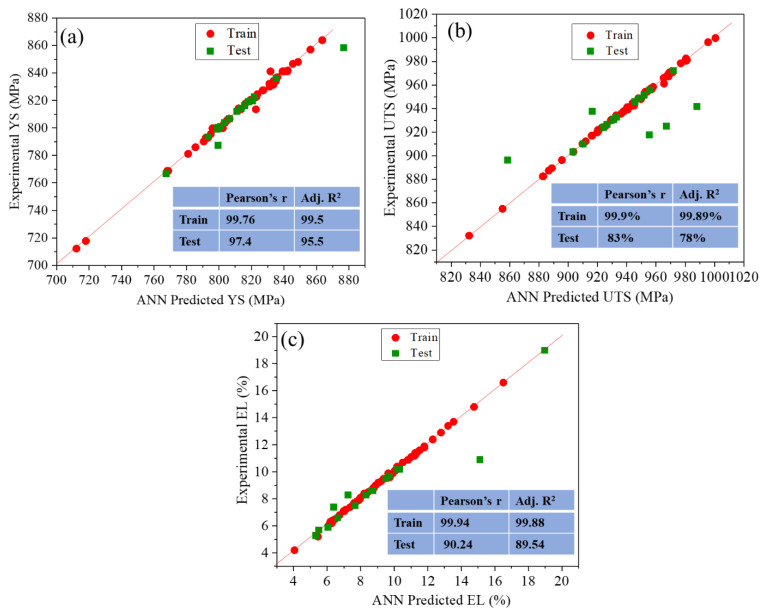
Validation of ANN model. (**a**) Model-1 YS, (**b**) Model-2 UTS, (**c**) Model-3 EL(%).

In neural systems, training is a process of updating an internal representation of an external system. The ANN model architecture with an input layer, hidden layers with hidden neurons, and an output layer is shown in Figure 2. The present ANN model is trained with the backpropagation algorithm with a sigmoid activation function [33]. The training program and graphical user interface were designed in C and java program, respectively. The training of the model considers the minimum difference in the predicted and experimental output values. The data were divided into training and testing datasets. The training datasets were used to develop the model, whereas the testing datasets were used to validate the model. The model hyperparameters such as the number of hidden layers, neurons in the hidden layers, momentum term, learning rate, and the number of iterations were optimized during training and testing to obtain an optimum model structure. The training procedure of the ANN model is shown in our previous study [31]. These hyperparameters were selected based on the minimum root mean square error (RMSE) and minimum average deviation in output prediction (E_tr_) of the test datasets, as shown in Figure 3. The following equation is used to calculate the RMSE of training [34].

(3) $$RMSE=\frac{1}{P}\sum_{p}\sum_{i}{\left(T_{ip}-O_{ip}\right)}^{2}$$

(4) $$E_{tr}(y)=\frac{1}{N}\sum_{i=1}^{N}\left|T_{i\;}\left(y\right)-O_{i}(y)\right|$$

where $E_{tr}(y)$ is the average error in the prediction by the testing datasets of the output parameters y, N = number of datasets, $T_{i}(y)$ = target output, and $O_{i}(y)$ = calculated output.

Since there are three different outputs in the original datasets, to obtain a high prediction of output values, we trained three independent models for each output parameter. The three independent models were developed with corresponding output YS, UTS, and %EL values. The optimum hyperparameters for all three models are shown in Table 1.

To assess the predictive accuracy of the developed ANN models, we report key performance metrics, including the root mean square error (RMSE) and the minimum average deviation in output prediction (Etr). Table 1 presents the ANN hyperparameters and accuracy indicators for YS, UTS, and %El, highlighting the optimized learning rates, momentum terms, and network architectures used in this study. The variation in the number of iterations for different mechanical properties is attributed to the complexity of their underlying relationships with microstructural parameters. The model for UTS required the highest number of iterations (17,346), indicating a more intricate dependency on microstructural features, while YS and %El converged with 13,254 and 15,561 iterations, respectively. The low RMSE values confirm the models’ effectiveness in capturing the complex relationships between microstructural parameters and mechanical properties.

## 3. Results and Discussion

### 3.1. Validation of ANN Model and Weights Distribution

The developed model performance was evaluated by Pearson’s r and Adj. R^2^ values between the experimental and predicted output values. The correlation between experimental and predicted values is shown in Figure 3. The values of the correlation coefficients of the training and test datasets indicate that the model predictions are comparably similar to the experimental data. The discrepancy in accuracy between the training and test datasets in Figure 3b is primarily due to the inherent complexity and variability of the dataset, as well as the limited number of test samples. While the training data (red circles) show an excellent correlation with experimental values, the test data (green squares) exhibit more scatter, particularly at higher UTS values, leading to a lower Pearson’s r (83%) and Adjusted R^2^ (78%) compared to the training set (99.9% and 99.89%, respectively).

This difference arises because the ANN model optimizes its internal parameters based on the training data, which results in a higher correlation. The test dataset, being independent, naturally introduces some deviations due to variations not captured during training. However, despite this discrepancy, the test predictions remain within an acceptable range and follow the overall trend, confirming the model’s reliability.

**Figure 4:**
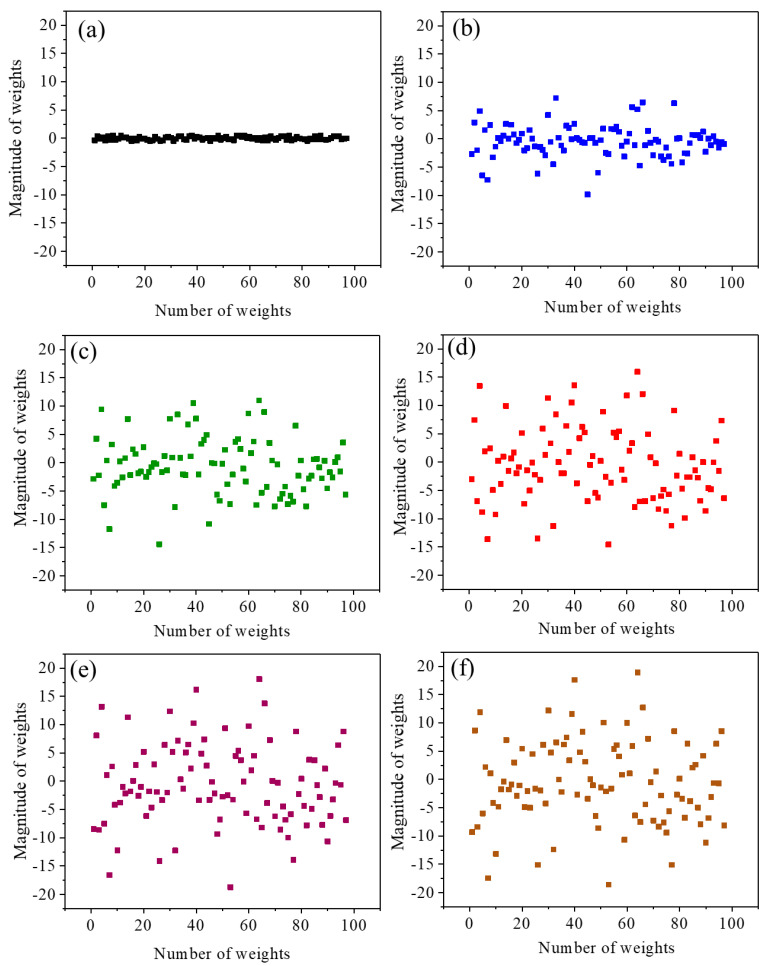
Weight distribution as a function of iterations. (**a**) Initial (0 iteration), (**b**) 5000, (**c**) 10,000, (**d**) 20,000, (**e**) 50,000, (**f**) 100,000.

The weights in the ANN model relate to the inputs and outputs and contain information about the relationship between the input and output parameters. Each neuron connected with different weights indicates the strength between the input and output data. Model training involves optimizing the weights for better prediction. The magnitude and the direction of the weights are responsible for the correlation between input and output variables [31]. The weights distribution as a function of iteration is shown in Figure 4. As we can see in Figure 4a, at the initial iterations, the weight is distributed within the magnitude range −0.5 to 0.45. As we increase the number of iterations, the magnitude of the weights increases to a considerable range (Figure 4b–e). Weights distributed in a wide range of magnitudes can improve the predictive performance of the model. At the optimum iteration, the magnitude of weights (from −20 to 20) remains constant, as shown in Figure 4e,f.

**Figure 5:**
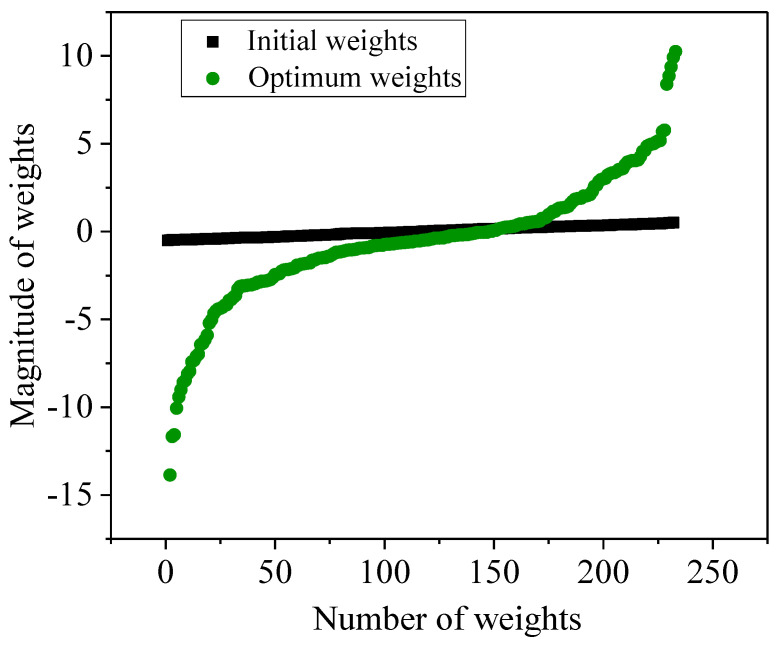
Formation of sigmoid activation function of weights.

The nature of weight distribution at different magnitudes with iteration is shown in Figure 5. The sigmoid activation forms at optimum weights, and at the initial iteration, the weights follow a straight line. Therefore, well-distributed weights are responsible for better predictions of unknown datasets.

### 3.2. Prediction of the Relationship Between Process Parameters and Mechanical Properties

**Figure 6:**
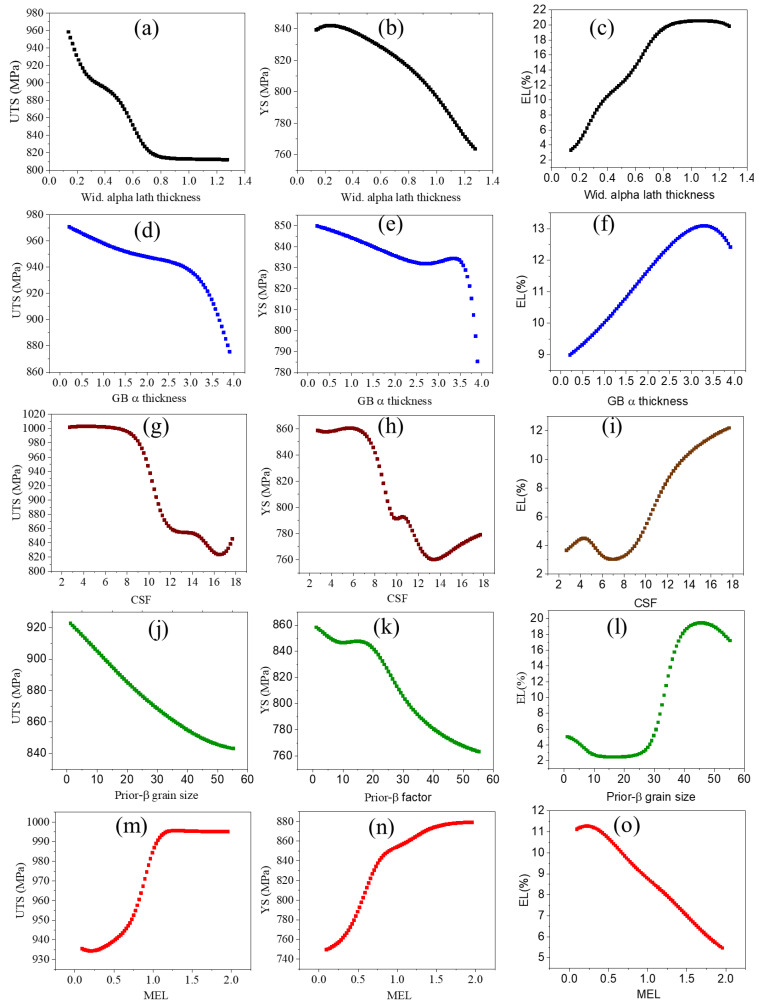
Relationships of ANN predicted outputs (UTS, YS, and %EL) to microstructural characteristics, shown as 2D plots.

The predicted outputs UTS, YS, and %EL are shown as a line plot in Figure 6 as a function of input variables. The UTS and YS plots are nearly similar in trend, as shown in Figure 6. As shown in Figure 6a,b, the UTS and YS decreased as Wida increased. The %EL increased with increasing Wida (Figure 6c) and GBα lath thickness (Figure 6f). This mechanism can be explained in terms of slip length and dislocation motion. The crack propagation behavior depends on the slip length. It is considered that the width of α lamellae determines the effective slip length, and in the martensitic type microstructure, the slip length is equal to the width of individual alpha plates. The crack propagation and dislocation motion can be restricted by fine alpha lath thickness and increases in the effective slip length [35]. In other words, the martensitic plates are strong obstacles to microcrack propagation. Therefore, the strength will always improve with decreasing α lath thickness [36]. The α plate dimension depends on the cooling rate; a high cooling rate leads to a reduction in α plate thickness as well as in colony size [37]. The decrease in the colony size decreases the slip length and increases the yield stress (Figure 6g–i). Thus, colony size (CS) strongly depends on Wida, as represented in the following equation [32].

CS = 6.7498W + 3.6683(5)

This equation shows that a smaller colony size provides additional constraints that restricted α lath growth. Lutjering [36] reported that with decreasing α colony size (slip length) the propagation rate of microcracks was decreased, with improved crack propagation resistance and ductility. However, a small α colony size is not effective for macrocrack propagation resistance, and for macrocrack propagation resistance and fracture toughness, a large α colony size is desirable. Lee et. al. [38] also reported the relationship between colony size and spacing between lamellae to mechanical properties. This study shows that increasing colony size and spacing between lamellae is associated with a decrease in yield strength, UTS and %EL. Tiley et. al. [39] reported that the α laths with a low aspect ratio exhibited increased ductility (at low temperatures), increased fatigue crack initiation resistance, and elevated temperature flow characteristics. The larger β grain provides more grain boundary α formation, which reduces the volume fraction of available α for lath growth, which in turn, increases the mean edge length. Thus, the prior-β grain size plot (Figure 6j–l) trends are similar to the GB α thickness plot (Figure 6d–f). The prior-β grain factor (PB) and mean edge length (MEL) can be drawn by the following equation [32].

PB = −8.5209M^3^ + 37.968M^2^ − 59.826M + 35.734 (6)

where PB refers to prior β grain size and M represents mean edge length (MEL). The mean edge length is expected to increase with decreasing Widmanstatten α lath thickness. The Widmanstatten α lath thickness and mean edge length can be correlated by the following relationship.

W = −0.6633M + 0.8634 (M < 0.7) W = 0.1008M^2^ − 0.3547M + 0.53 (M > 0.7) (7)

The effects of mean edge length on UTS, YS, and %EL are shown in Figure 6m–o, respectively. With increasing mean edge length, the UTS, as well as YS, increases and %EL decreases. The mean edge length plot trend is exactly opposite that of the Widmanstatten α lath thickness plot (Figure 6a–c) due to interrelationship.

The β grain size affects the Widmanstatten α plate width. This shows that a larger β grain size is associated with a decrease in the α plate width of the Widmanstatten colonies [37]. This is possible due to the increase in the β grain size with decreases in the transformation temperature of β→α [40]. Thus, the diffusivity of elements to form the α phase decreases with increasing β grain size. The large grain size, considering the Hall Petch relation of grain boundary strengthening, could be interpreted as an adverse effect on strength (Figure 6j–l).

**Figure 7:**
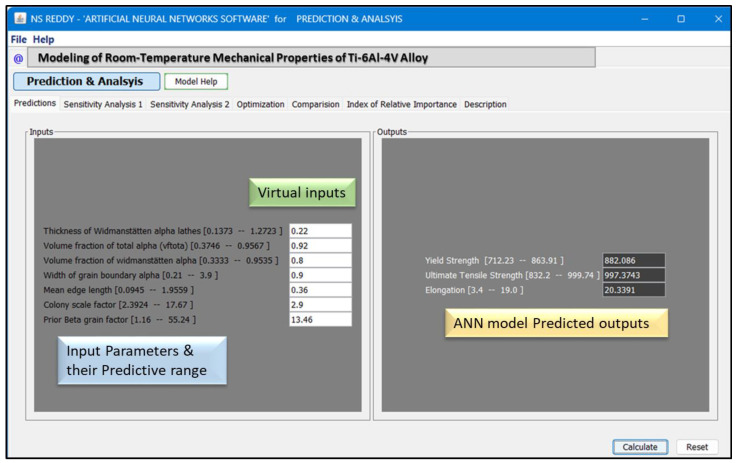
GUI of the developed model for predicting UTS.

We developed a stand-alone graphical user interface (GUI) for easy use of the model. We can calculate the mechanical properties with the help of the GUI by incorporating the microstructural parameters (characteristics) in the input window, as shown in Figure 7 for UTS.

### 3.3. Graphical User Interface

The graphical user interface (GUI) shown above is part of an artificial neural network (ANN) software package developed to model the relationship between the microstructural features and mechanical properties of Ti-6Al-4V alloys. The interface is designed to allow users to perform predictions and analyses efficiently based on specific input parameters. The left section of the GUI represents the input parameters, divided into two systems: the predictive range and the virtual input system. The predictive range, highlighted in the blue box, shows the allowable ranges for input parameters such as Widmanstatten α lath thickness (wida), Widmanstätten α volume fraction (Volα), grain boundary α thickness (gbα), α colony size (αcol), and prior β grain size (priorβ). The virtual input system, highlighted in the red box, allows users to input specific values within the predictive range for analysis and prediction.

The right section of the GUI provides the predicted mechanical properties generated by the ANN model. In this example, the model predicts the mechanical properties of the Ti-6Al-4V alloy based on the mean values of microstructural features. The predicted properties are displayed numerically for easy interpretation, enabling users to assess the impact of microstructural variations on mechanical properties. The GUI also includes interactive features such as a “Calculate” button, which computes the predicted output based on the entered values, and a “Reset” button to clear the inputs for a new analysis.

To further validate the model’s reliability, we used the GUI to predict mechanical properties for a new set of input values based on the mean of all input parameters, which are not experimental values but serve as a representative dataset. The predictions obtained were metallurgically meaningful and within the expected range, reinforcing the accuracy and applicability of the ANN model. This additional verification step ensures that the model generalizes well to unseen data, making it a valuable tool for optimizing heat treatment strategies and improving the performance of Ti-6Al-4V alloys.

### 3.4. Index of the Relative Importance

**Figure 8:**
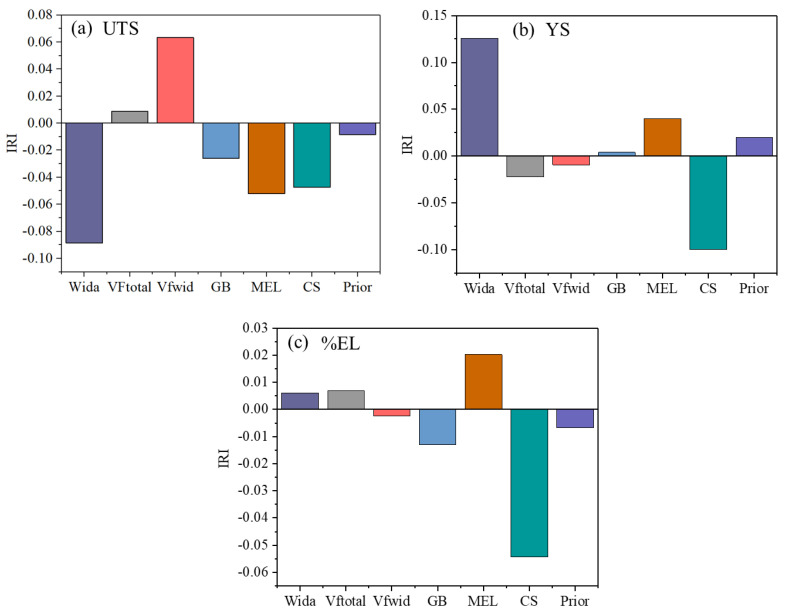
The index of the relative importance of microstructural features on (**a**) UTS, (**b**) YS, and (**c**) %EL.

The effects of microstructural characteristics on the mechanical properties of Ti-6Al-4V alloys were studied with the help of the index of relative importance (IRI). The IRI is a vector quantity; the magnitude and the direction of the IRI indicate the importance of the input variables on the output process variables. A higher value of IRI for an input parameter indicates a stronger degree of relationship with the output parameter [30]. A negative value of IRI indicates that the corresponding input parameters have an adverse effect on the output parameters. In this study, we consider a 10% band (+5% to −5%) for each input variable. The IRI is calculated for every individual dataset, and the average values of IRI for UTS and YS are shown in Figure 8a and Figure 8b, respectively. Figure 8a shows that the volume fraction of Vfwid and MEL have a positive impact on UTS. This means that, with increasing Vfwid and MEL, the UTS increases, whereas Wida, GBα, and CSF show a negative effect on UTS. A similar effect can be seen in Figure 8b for YS. The effect of VFtotal, volume fraction of Vfwid, GBα, and prior β on YS is much less.

This study introduces several novel aspects in applying artificial neural networks (ANNs) to model the relationship between microstructural parameters and mechanical properties of Ti-6Al-4V alloys. The ANN model effectively captures the non-linear relationships between microstructural features and mechanical behavior, providing a more accurate alternative to conventional regression-based methods. To enhance interpretability, we incorporated the index of relative importance (IRI), which quantifies the contribution of each microstructural parameter to the mechanical properties, offering valuable insights for heat treatment optimization. Additionally, a custom-built graphical user interface (GUI) allows researchers and engineers to predict mechanical properties from microstructural data without requiring programming expertise, facilitating real-time analysis and practical application.

## 4. Conclusions

1. In this study, an ANN model was developed to study the influence of microstructural parameters on the mechanical properties of Ti-6Al-4V alloys. The developed model shows a high Adj. R^2^ value for the training and testing datasets for UTS, YS, and %EL. This shows that the model is highly accurate, with predictions of output values near the experimental values.
2. The effects of microstructural parameters on the mechanical properties of Ti-6Al-4V alloys was explained with the help of 2D plots. The model’s predicted results show that the UTS of Ti-6Al-4V decreases with increases in prior β grain size, Widmanstatten α lath thickness, grain boundaries α thickness, colony scale factor, while UTS increases with mean edge length.
3. The significance of each microstructural parameter on mechanical properties was described with the help of an index of relative importance. The Wida, Vfwid, MEL, and CSF are the parameters which most affect UTS.

## Figures and Tables

**Table 1:** Optimum hyperparameters of the developed model.

Model	Learning Rate	Momentum Term	Iterations	Hidden Layers and Respective Neurons in Hidden Layer	RMSE	E_tr_
Model-1 (YS)	0.5	0.6	13,254	2 9 9	0.00001	0.675
Model-2 (UTS)	0.6	0.7	17,346	2 6 6	0.00001	3.42
Model-3 (%EL)	0.7	0.5	15,561	2 6 6	0.00001	0.268

## Data Availability

The original contributions presented in this study are included in the article/Supplementary Material. Further inquiries can be directed to the corresponding authors.

## Supplementary Materials

The following supporting information can be downloaded at: https://www.mdpi.com/article/10.3390/ma18051099/s1, Table S1: The Actual data used for ANN Modeling; Table S2: Minimum and Maximum values of the data resulted from the Normalization program. Table S3: Normalized inputs and output variables of the data resulted from the Normalization program.
