# Supplementary material for: Artificial Neural Network Modeling of Ti-6Al-4V Alloys to Correlate Their Microstructure and Mechanical Properties
Source: Materials (Basel). 2025 Feb 28;18(5):1099. doi: 10.3390/ma18051099 (PMC11901288; doi:10.3390/ma18051099)
Supplement: Supplementary file 1 [file materials-18-01099-s001.zip › materials-3457555-supplementary.pdf]

Supplementary Material

Artificial Neural Network Modeling of Ti-6Al-4V Alloys to Correlate Their Microstructure and Mechanical Properties

A. K. Maurya [1,2,†], P. L. Narayana [1,†], Jong-Taek Yeom [1,*], Jae-Keun Hong [1] and N. S. Reddy [2,*]

[1] Lightweight Materials Research Division, Korea Institute of Materials Science, Changwon 51508, Republic of Korea; jkhong@kims.re.kr (J.-K.H.)
[2] Virtual Materials Lab, School of Materials Science and Engineering, Engineering Research Institute, Gyeongsang National University, Jinju 52828, Republic of Korea
* Correspondence: yjt96@kims.re.kr (J.-T.Y.); nsreddy@gnu.ac.kr (N.S.R.)
† These authors contributed equally to this work.

The C program used in normalization of the data.

```
#include <stdio.h>
#include <stdlib.h>
void main()
{
	FILE *fp1,*fp2,*fp3;
	int i,j,k,io,nsamp,inp,out;
	float *a,*min,*max,*norm ;
	char name1[20],name2[20],name3[20];
	printf("Enter task(INPUT) file name\n");
	scanf("%s",name1);
	printf("Enter MINMAX file name \n");
	scanf("%s",name2);
	printf("Enter NORMALISED file name \n");
	scanf("%s",name3);
	fp1 = fopen(name1,"r") ;
	printf("enter no. of inputs, outputs & no. of sampals \n");
	scanf("%d %d %d",&inp,&out,&nsamp);
	io = inp + out;
	a = (float*) calloc (io+2,sizeof(float)) ;
	norm = (float*) calloc (io+2,sizeof(float)) ;
	min = (float*) calloc (io+2,sizeof(float)) ;
```

```
        max = (float*) calloc (io+2,sizeof(float)) ;
        for(j=0;j<nsamp;j++)   {
                for(i=0;i<io;i++) {
                        fscanf(fp1,"%f",&a[i]);
                        if(0==j) { min[i] = a[i] ; max[i] = a[i] ; }
                        if(min[i] > a[i] ) min[i] = a[i] ;
                        if(max[i] < a[i] ) max[i] = a[i] ;
                }
        }
        fclose(fp1);
/*      TO PRINT MIN. & MAX. COLOUMWISE IN A FILE              */

        fp2=fopen(name2,"w") ;
        for(i=0;i<io;i++) {
                fprintf(fp2,"%f\t",min[i]) ;
                printf("min = %f\n",min[i]) ;
        }
        fprintf(fp2,"\n") ;
        for(i=0;i<io;i++) {
                fprintf(fp2,"%f\t",max[i]) ;
                printf("max = %f\n",max[i]) ;
        }
        fprintf(fp2,"\n") ;
        fclose(fp2) ;
        fp1 = fopen(name1,"r") ;
        fp3 = fopen(name3,"w") ;
        for(j=0;j<nsamp;j++)      {
                for(i=0;i<io;i++) {
                        fscanf(fp1,"%f",&a[i]) ;
                        if((max[i] - min[i]) != 0)  {
                                norm[i] = ((a[i] - min[i])/((max[i] - min[i])/.8)) +.1 ;
                                fprintf(fp3,"%f\t",norm[i]);
                        }
                }
```

```
            fprintf(fp3,"\n");
        }
        fclose(fp1) ;
        fclose(fp3) ;
}
```

Supplementary Table S1. The Actual data used for ANN Modeling

Sample	Wida	Vftota	Vfwid	GB	mel	cs	Prior	YS	UTS	Elong
1	0.1445	0.9022	0.9006	1.5	1.9559	10.21	31.67	806.69	930.79	7.4
2	0.243	0.875	0.8029	1.45	0.6553	5.3	29.38	834.27	937.69	7.9
3	1.077	0.9295	0.8494	1.58	0.1428	8.8	29.62	813.58	882.53	9.7
4	0.2866	0.9247	0.8734	1.08	0.8481	5.57	47.03	841.16	930.79	10.3
5	0.2834	0.8782	0.8381	1.64	0.7304	5.24	19.41	813.58	923.90	8.4
6	0.3769	0.9335	0.8998	0.92	0.801	4.9	49.24	841.16	917.00	11.1
7	0.1373	0.891	0.8205	1.04	1.5498	5.63	32.99	806.69	958.37	9
8	0.2119	0.9183	0.8349	1.32	1.2183	10.62	21.29	834.27	944.58	10.1
9	0.5905	0.9279	0.8798	3.01	0.1567	16.13	11.9	841.16	896.32	8.5
10	0.4459	0.9327	0.8718	1.22	0.3896	15.36	31.43	799.79	910.11	9.5
11	0.3698	0.9183	0.8317	1.49	0.4961	13.96	18.86	786.00	854.95	8
12	0.6319	0.9247	0.883	1.53	0.211	16.89	21.45	799.79	882.53	8
13	0.2355	0.8942	0.8942	0.87	1.3229	5.83	13.87	834.27	937.69	4.2
14	0.2325	0.859	0.8077	1.23	0.6604	10.79	13.01	827.37	930.79	7.1
15	1.0992	0.9183	0.867	1.59	0.202	12.27	25.93	820.48	882.53	6.3
16	0.3702	0.9087	0.8798	2.59	0.701	6.02	26	792.90	910.11	11.4
17	0.2889	0.9054	0.8702	1.66	0.6908	5.17	52.56	834.27	930.79	10.7
18	0.3563	0.9167	0.8638	1.35	0.6908	5.58	38.65	827.37	917.00	10.9
19	0.3345	0.8734	0.8173	1.9	0.4395	11.36	31.11	813.58	944.58	6.4
20	0.4678	0.8928	0.8189	2.02	0.271	11.99	32.6	841.16	937.69	7.1
21	1.2723	0.9503	0.8894	1.41	0.0945	17.67	20.29	841.16	903.21	7.1
22	0.4655	0.9071	0.8702	0.91	0.4169	11.38	18.7	792.90	889.42	10.9
23	0.488	0.9455	0.8109	1.82	0.3003	7.32	41.82	834.27	903.21	11.5
24	0.4317	0.8798	0.758	0.98	0.34	6.44	55.24	806.69	923.90	8.8
25	0.2828	0.9071	0.8894	1.08	0.7446	5.15	37.09	841.16	930.79	11.9
27	0.2522	0.9247	0.8494	1.18	1.0774	4.4675	34.77	863.91	970.78	9.9
28	0.206	0.9199	0.9167	0.21	1.4131	4.7781	2.34	857.02	996.29	7.7
29	0.6083	0.9087	0.875	1.24	0.2353	8.8476	4.86	841.85	948.72	14.8
30	0.4465	0.9038	0.899	3.9	0.6929	3.5565	1.16	812.89	972.16	9.5
31	0.2601	0.9054	0.8903	1.44	0.9485	3.8855	2.75	806.00	978.37	8.3
32	0.9324	0.9263	0.859	1.88	0.2371	6.9106	22.91	768.77	896.32	19
33	0.5889	0.9103	0.9048	2.97	0.322	6.95	1.54	795.65	932.86	6.6
34	0.414	0.8766	0.8675	1.38	0.734	8.7107	1.89	830.13	981.12	8.6
35	0.4331	0.9375	0.9358	1.74	0.6127	7.047	1.35	814.27	965.96	10.4
36	0.2953	0.9551	0.9499	1.56	0.5681	6.8293	2.35	843.23	999.74	6.8

37	0.2689	0.9375	0.932	1.59	0.807	7.075	1.62	820.48	961.13	5.2
38	0.4842	0.9199	0.8574	3.12	0.2475	12.0999	6.76	821.86	930.10	11.6
39	0.3332	0.9423	0.3394	1.31	0.633	8.5157	18.62	803.93	934.24	13.4
40	0.2567	0.9343	0.9214	3.21	0.774	4.3528	5.63	785.31	980.43	8.3
41	0.7643	0.9375	0.9215	2.7	0.153	11.6056	2.61	832.20	919.76	10.1
42	0.578	0.9119	0.3713	2.59	0.2484	11.2012	11.47	831.51	967.33	11.3
43	0.5424	0.3746	0.3451	2.35	0.263	6.3278	15.21	837.02	954.23	9.2
44	0.7734	0.9455	0.9279	2.34	0.1775	10.5859	11.12	812.20	921.14	13.7
45	0.5431	0.9183	0.9114	1.16	0.4129	3.4562	1.86	814.27	956.30	3.4
46	0.5462	0.9247	0.3691	1.49	0.3429	3.405	26.09	766.70	887.36	15.6
47	0.33359	0.9231	0.3608	2.63	0.172	3.2105	23.5	806.69	935.62	12.9
48	0.2668	0.9135	0.9119	1.49	0.3454	7.2593	1.85	824.61	970.78	7.8
49	0.5474	0.3766	0.3401	1.93	0.308	3.0573	25.21	850.12	954.23	13.3
50	0.3954	0.9567	0.9535	1.51	0.6919	5.4483	1.27	863.91	970.78	9.9
51	0.5249	0.835	0.3642	2.91	0.305	3.2479	13.49	717.74	912.18	8.1
52	0.7009	0.9083	0.853	2.54	0.238	9.6111	19.42	801.86	925.28	12.4
53	0.9222	0.3917	0.3795	2.47	0.225	9.5524	2.81	712.23	832.20	6.5
54	0.4644	0.915	0.3333	2.37	0.33	9.03	10.37	817.72	948.72	7.2
55	0.4134	0.9051	0.8395	1.19	0.601	8.5813	4.23	768.77	941.82	5.2
56	0.5004	0.9439	0.9233	1.79	0.3591	9.5083	2.34	803.24	967.33	11.3
57	0.2542	0.9231	0.9199	0.71	1.1666	5.9469	1.37	790.14	942.51	9.6
59	0.5039	0.383	0.8761	1.57	0.331	6.2294	2.53	793.59	948.03	5.9
61	0.313	0.9375	0.9271	1.33	0.91	7.1733	3.35	815.65	982.50	5.7
62	0.2202	0.9119	0.399	0.97	1.5034	6.6426	1.45	819.79	919.07	3.6
63	0.3175	0.8933	0.8723	1.34	0.926	4.0624	11.53	799.10	941.82	7.5
64	0.393	0.9333	0.8951	1.47	0.566	4.435	23.38	836.33	917.00	10.2
65	0.3672	0.9433	0.9103	1.3	0.742	5.0579	22.75	816.34	930.10	9.5
66	0.4169	0.8966	0.8411	1.72	0.182	5.0501	18.91	793.59	925.28	9.3
67	0.5376	0.9151	0.9116	1.51	0.519	3.911	5.06	803.93	926.66	9.2
68	0.4957	0.9231	0.9092	0.33	0.675	3.4671	6.99	822.54	939.07	11.2
69	0.2463	0.9311	0.9016	1.47	0.36	3.8683	24.13	781.18	921.14	6
70	0.3536	0.9455	0.9212	1.67	0.33	3.4401	19.3	822.54	917.69	5.3
71	0.2182	0.9215	0.3939	0.91	0.36	2.3924	13.46	848.06	969.40	10.2
72	0.2335	0.9135	0.3666	0.95	0.612	3.254	17.17	819.79	921.83	9.7
73	0.3908	0.9183	0.3766	0.39	0.255	2.7167	12.1	794.28	934.24	10.9
74	0.2269	0.9247	0.9038	0.98	0.489	2.8009	18.26	858.40	919.76	8.3
75	0.2332	0.9279	0.9227	1.78	0.389	3.0718	7.79	787.38	945.96	7.4

Supplementary Table S2. Minimum and Maximum values of the data resulted from the Normalization program.

	Wida	Vftota	Vfwid	GB	mel	cs	Prior	YS	UTS	Elong
**Minimum**	0.1373	0.3746	0.3333	0.21	0.0945	2.3924	1.16	712.23	832.2	3.4
**Maximum**	1.2723	0.9567	0.9535	3.9	1.9559	17.67	55.24	863.91	999.74	19

Supplementary Table S3. Normalized inputs and output variables of the data resulted from the Normalization program.

Sample	Wida	Vftota	Vfwid	GB	mel	cs	Prior	YS	UTS	Elong
1	0.105	0.825	0.832	0.380	0.900	0.509	0.551	0.598	0.571	0.305
2	0.175	0.788	0.706	0.369	0.341	0.252	0.517	0.744	0.604	0.331
3	0.762	0.863	0.766	0.397	0.121	0.436	0.521	0.635	0.340	0.423
4	0.205	0.856	0.797	0.289	0.424	0.266	0.779	0.780	0.571	0.454
5	0.203	0.792	0.751	0.410	0.373	0.249	0.370	0.635	0.538	0.356
6	0.269	0.868	0.831	0.254	0.404	0.231	0.811	0.780	0.505	0.495
7	0.100	0.810	0.728	0.280	0.725	0.270	0.571	0.598	0.702	0.387
8	0.153	0.847	0.747	0.341	0.583	0.531	0.398	0.744	0.637	0.444
9	0.419	0.860	0.805	0.707	0.127	0.819	0.259	0.780	0.406	0.362
10	0.318	0.867	0.795	0.319	0.227	0.779	0.548	0.562	0.472	0.413
11	0.264	0.847	0.743	0.378	0.273	0.706	0.362	0.489	0.209	0.336
12	0.449	0.856	0.809	0.386	0.150	0.859	0.400	0.562	0.340	0.336
13	0.169	0.814	0.824	0.243	0.628	0.280	0.288	0.744	0.604	0.141
14	0.167	0.766	0.712	0.321	0.343	0.540	0.275	0.707	0.571	0.290
15	0.778	0.847	0.788	0.399	0.146	0.617	0.466	0.671	0.340	0.249
16	0.264	0.834	0.805	0.616	0.361	0.290	0.467	0.525	0.472	0.510
17	0.207	0.829	0.793	0.414	0.356	0.245	0.860	0.744	0.571	0.474
18	0.254	0.845	0.784	0.347	0.356	0.267	0.655	0.707	0.505	0.485
19	0.239	0.786	0.724	0.466	0.248	0.570	0.543	0.635	0.637	0.254
20	0.333	0.812	0.726	0.492	0.176	0.603	0.565	0.780	0.604	0.290
21	0.900	0.891	0.817	0.360	0.100	0.900	0.383	0.780	0.439	0.290
22	0.331	0.832	0.793	0.252	0.239	0.571	0.359	0.525	0.373	0.485
23	0.347	0.885	0.716	0.449	0.188	0.358	0.701	0.744	0.439	0.515
24	0.308	0.794	0.648	0.267	0.206	0.312	0.900	0.598	0.538	0.377
25	0.203	0.832	0.817	0.289	0.379	0.244	0.632	0.780	0.571	0.536
27	0.181	0.856	0.766	0.310	0.522	0.209	0.597	0.900	0.762	0.433
28	0.148	0.849	0.853	0.100	0.667	0.225	0.117	0.864	0.884	0.321
29	0.432	0.834	0.799	0.323	0.161	0.438	0.155	0.784	0.656	0.685
30	0.318	0.827	0.830	0.900	0.357	0.161	0.100	0.631	0.768	0.413
31	0.187	0.829	0.818	0.367	0.467	0.178	0.124	0.595	0.798	0.351
32	0.660	0.858	0.778	0.462	0.161	0.337	0.422	0.398	0.406	0.900
33	0.418	0.836	0.837	0.698	0.198	0.339	0.106	0.540	0.581	0.264
34	0.295	0.790	0.789	0.354	0.375	0.431	0.111	0.722	0.811	0.367
35	0.308	0.874	0.877	0.432	0.323	0.344	0.103	0.638	0.739	0.459
36	0.211	0.898	0.895	0.393	0.304	0.332	0.118	0.791	0.900	0.274

37	0.193	0.874	0.872	0.399	0.406	0.345	0.107	0.671	0.716	0.192
38	0.345	0.849	0.776	0.731	0.166	0.608	0.183	0.678	0.567	0.521
39	0.238	0.880	0.108	0.338	0.331	0.421	0.358	0.584	0.587	0.613
40	0.184	0.869	0.859	0.750	0.392	0.203	0.166	0.485	0.808	0.351
41	0.542	0.874	0.859	0.640	0.125	0.582	0.121	0.733	0.518	0.444
42	0.411	0.838	0.149	0.616	0.166	0.561	0.253	0.729	0.745	0.505
43	0.386	0.100	0.115	0.564	0.172	0.306	0.308	0.758	0.683	0.397
44	0.548	0.885	0.867	0.562	0.136	0.529	0.247	0.627	0.525	0.628
45	0.386	0.847	0.846	0.306	0.237	0.156	0.110	0.638	0.693	0.100
46	0.388	0.856	0.146	0.378	0.207	0.153	0.469	0.387	0.363	0.726
47	0.238	0.854	0.135	0.625	0.133	0.143	0.430	0.598	0.594	0.587
48	0.191	0.841	0.846	0.378	0.208	0.355	0.110	0.693	0.762	0.326
49	0.389	0.103	0.109	0.473	0.192	0.135	0.456	0.827	0.683	0.608
50	0.282	0.900	0.900	0.382	0.357	0.260	0.102	0.900	0.762	0.433
51	0.373	0.733	0.140	0.685	0.190	0.145	0.282	0.129	0.482	0.341
52	0.497	0.833	0.770	0.605	0.162	0.478	0.370	0.573	0.544	0.562
53	0.653	0.124	0.160	0.590	0.156	0.475	0.124	0.100	0.100	0.259
54	0.331	0.843	0.100	0.568	0.201	0.448	0.236	0.656	0.656	0.295
55	0.295	0.829	0.753	0.312	0.318	0.424	0.145	0.398	0.623	0.192
56	0.356	0.882	0.861	0.443	0.214	0.473	0.117	0.580	0.745	0.505
57	0.182	0.854	0.857	0.208	0.561	0.286	0.103	0.511	0.627	0.418
59	0.358	0.112	0.800	0.395	0.202	0.301	0.120	0.529	0.653	0.228
61	0.224	0.874	0.866	0.343	0.450	0.350	0.132	0.645	0.818	0.218
62	0.158	0.838	0.185	0.265	0.706	0.323	0.104	0.667	0.515	0.110
63	0.227	0.813	0.795	0.345	0.457	0.187	0.253	0.558	0.623	0.310
64	0.280	0.868	0.825	0.373	0.303	0.207	0.429	0.755	0.505	0.449
65	0.262	0.882	0.844	0.336	0.378	0.240	0.419	0.649	0.567	0.413
66	0.297	0.817	0.755	0.427	0.138	0.239	0.363	0.529	0.544	0.403
67	0.382	0.843	0.846	0.382	0.282	0.180	0.158	0.584	0.551	0.397
68	0.353	0.854	0.843	0.126	0.349	0.156	0.186	0.682	0.610	0.500
69	0.177	0.865	0.833	0.373	0.214	0.177	0.440	0.464	0.525	0.233
70	0.252	0.885	0.858	0.417	0.201	0.155	0.368	0.682	0.508	0.197
71	0.157	0.852	0.178	0.252	0.214	0.100	0.282	0.816	0.755	0.449
72	0.168	0.841	0.143	0.260	0.322	0.145	0.337	0.667	0.528	0.423
73	0.279	0.847	0.156	0.139	0.169	0.117	0.262	0.533	0.587	0.485
74	0.163	0.856	0.836	0.267	0.270	0.121	0.353	0.871	0.518	0.351
75	0.168	0.860	0.860	0.440	0.227	0.136	0.198	0.496	0.643	0.305
